# Autophagy induced by *H. pylori* VacA regulated the survival mechanism of the SGC7901 human gastric cancer cell line

**DOI:** 10.1007/s13258-021-01151-7

**Published:** 2021-08-16

**Authors:** Juan Luo, Luyan Bai, Jun Tao, Yu Wen, Mingke Li, Yunzhen Zhu, Sufeng Luo, Guangyu Pu, Lanqing Ma

**Affiliations:** 1 Department of Gastroenterology, Yunnan Province Clinical Research Center for Digestive Diseases, The First Affiliated Hospital of Kunming Medical University, Kunming Medical University, 295 Xichang Road, Kunming, 650032 China; 2 The People’s Hospital of Mengzi, Mengzi, China; 3 Technology Transfer Center, Kunming Medical University, Kumming, China

**Keywords:** *Helicobacter pylori*, Vacuolating cytotoxin, Reactive oxygen species, Autophagy

## Abstract

**Background:**

Vacuolating cytotoxin (VacA) is an important virulence factor of *Helicobacter pylori* (*H. pylori*). It was previously believed that VacA can trigger the cascade of apoptosis on mitochondria to lead to cell apoptosis. Recently, it was found that VacA can induce autophagy. However, the molecular mechanism by which VacA induces autophagy is largely unknown.

**Objective:**

We aimed to explore the molecular mechanism of autophagy induced by *H. pylori* in gastric cancer cells and the effect of autophagy on the survival of gastric cancer cells.

**Methods:**

The autophagy of human gastric cancer cell line SGC7901 was detected by Western blot and RT-PCR in the treatment of VacA protein of *H. pylori*. The relationship between autophagy and reactive oxygen species (ROS) in the proliferation of gastric cancer cells were studied by gene expression silences (siRNA) and CM-H2DCFDA (DCF) staining.

**Results:**

The results showed that VacA protein secreted by *H. pylori* in the supernatant stimulated autophagy in SGC7901 cells. After VacA protein treatment, the mRNA expressions of BECN1, ATG7 and PIK3C3, were up-regulated. ATG7 silencing by siRNA inhibited VacA-induced autophagy. Furthermore, our data demonstrated that VacA protein increased ROS levels. Addition of the antioxidant *N*-acetyl-l-cysteine (NAC) suppressed the levels of ROS, leading to inhibition of autophagy.

**Conclusions:**

*H. pylori* VacA is a key toxin that induces autophagy by increased ROS levels. And our findings demonstrated that VacA significantly inhibited proliferation in SGC7901 cells.

## Introduction

Helicobacter pylori (*H. pylori*), a spiral-shaped, curved, gram staining-negative bacterium, is one of the most common pathogens in the human body. *H. pylori* infection can cause a wide range of gastric diseases, including gastritis, peptic ulcer, and gastric cancer (Diaconu et al. 2017). It has been shown that at least 75% of gastric cancer cases are due to *H. pylori* infection (Plummer et al. 2015; Hartgrink et al. 2009). Because of its close association with gastric cancer, *H. pylori* is classified as a class I carcinogen by the International Organization for Research on Cancer in 1994 (Loomis et al. 2014).

*H. pylori* can secrete many virulence factors, including urease (Ure), cytotoxin-associated gene A (CagA) and vacuolating cytotoxin A (VacA). VacA, one of the most important virulence factors in *H. pylori*, is named vacuolating toxin because it causes cellular vacuolar degeneration. The VacA precursor is first encoded by the VacA gene to form VacA. During exocytic secretion of the VacA precursor, the amino- and carboxyl-terminal ends are processed and modified to form the approximately 88-kDa mature and active VacA (Loomis et al. 2014). The VacA has many pathogenesis, such as induction of vacuole formation, induction of apoptosis, and immune regulation function (Terebiznik et al. 2009; Ki et al. 2008; Bronte-Tinkew et al. 2009).

Autophagy, a highly conserved process in eukaryotes, can degrade aging and damaged organelles to obtain amino acids and other macromolecules for recycling, thus playing an important role in maintaining the stability of the internal environment of the body (Mizushima et al. 2008; Greenfield and Jones 2013). Current studies have shown that *H. pylori* infection can induce autophagy in gastric epithelial cells, which can degrade pathogens. VacA is indispensable in *H. pylori* infection-induced autophagy (Terebiznik et al. 2009; Halder et al. 2015). However, the molecular mechanism by which VacA mediates autophagy is still unclear. Some studies indicate that *H. pylori*-induced autophagy may be related to ROS (Tsugawa et al. 2012; Yahiro et al. 2015). It has been reported that *H. pylori* infection leads to an increase in ROS levels, which continue to increase with *H. pylori* infection (Calvino and Parra 2010). *H. pylori* infection causes an increase in ROS mainly through influencing mitochondrial function by VacA. VacA is inserted into the mitochondrial inner membrane to form a channel, thereby reducing the mitochondrial outer membrane potential and leading to mitochondrial destruction.

In the present study, we attempted to investigate the relationships among VacA, ROS levels and autophagy in a gastric cancer cell line (SGC7901). Here, we report that *H. pylori* VacA induces autophagy in gastric cancer cells through induction of ROS.

## Materials and methods

### Cell line and bacterial strain

*H. pylori* strain NCTC11639 was presented by Professor Chen Ye (Department of Gastroenterology, Southern Medical University, Guangzhou, China). The human gastric cancer cell line SGC790 was purchased from Beijing Zhonglei Biotechnology Co., Ltd.

### Antibodies and reagents

A GFP-LC3 plasmid was obtained from Professor Tamotsu Yoshimori (Department of Cell Biology, National Institute for Basic Biology, Presto, Japan). Antibodies against P62 (sc-25329), LC3II (sc-376404), VacA (sc-32746), and β-tubulin (sc-166729) were obtained from Santa Cruz Biotechnology. Some chemical reagents, including H2DCF-DA (HY-D0940) and NAC (HY-B0215), were purchased from MedChemExpress. The additional chemical reagents Tris and DMSO were purchased from Gibco-BRL and Sigma. An MTS kit was purchased from Promega (USA). siRNA (siATG7) was purchased from Shanghai Jima Co., Ltd. Primers were obtained from Invitrogen.

### RNA interference

Small interfering RNA (siRNA) for the knockdown of ATG7 (siATG7) was obtained from Shanghai GenePharma Co., Ltd. Control RNAi (Shanghai GenePharma Co., Ltd) was used as a negative control. The reverse transfection method was used to transfect siATG7 at a final concentration of 100 nM into SGC790 cells.

### Preparation of *H. pylori* culture supernatant

When the OD620 value was 1.5, the *H. pylori* Culture fluid was collected, centrifuged at 10,000 rpm for 15 min, and the supernatant was transferred to a new centrifuge tube. Pass through 0.22 μm filter head to remove any bacteria. The filtered supernatant was transferred to a 30kD ultrafiltration tube, centrifuged for 3500*g* for 15 min at 4 °C. The supernatant after ultrafiltration was diluted to 30 times with Ham's F-12 medium.

### Purification of VacA

The saturated ammonium sulfate solution was added to the *H. pylori* supernatant to get a final concentration of 40%, after which the beaker was placed on a magnetic suspension stirrer and stirred for 6 h. Then the precipitate was centrifuged at 10,000 rpm for 30 min. The collected precipitate was dissolved in PBS, put into a dialysis bag, stirred for 24 h at 4 °C and changed once every 6 h to completely remove the ammonium sulfate. The liquid in the dialysis bag was centrifuged at 12,000 rpm for 30 min, and the protein content in the supernatant was determined.

### Inactivation of VacA

The VacA within purified VacA preparations was removed by incubation for 2 h at 4 °C with Protein A magnetic beads (Cell Signaling) that had been preincubated for 4 h at 4 °C with anti-VacA antibody (Santa Cruz).

### In vitro* H. pylori* infection model

For infection assays, gastric cancer cells were infected with *H. pylori* suspension a multiplicity of infection (MOI) of 100:1 (bacteria: cell) as previously described (Valenzuela et al. 2010). The gastric cells incubated with concentrated culture supernatant at a final dilution of 1:25 as described by Valenzuela et al. (2013).

### Western blotting

Cells were lysed in radioimmunoprecipitation assay buffer and total proteins were extracted with protease inhibitor. SDS-PAGE gel electrophoresis and membrane transfer to PVDF membrane (Invitrogen). Primary antibody incubation were diluted in advance, and the closed PVDF membrane was placed in a 4 °C shaker of primary antibody for overnight incubation. Horseradish peroxidase labeled secondary antibody was selected according to the source of primary antibody. Detection of the target protein bands were developed by ECL method (GE Healthcare, Piscataway, NJ).

### Measurement of ROS levels

After the addition of VacA and 5 × CMCC and incubation for 8 h, gastric cancer cells were incubated in RPMI 1640 culture medium (Hyclone) containing 5 mM NAC (Invitrogen) for 1 h and washed three times with PBS. Then incubate the cells with 5 µM H2DCF-DA (Invitrogen) in dark at 37 °C for 1 h. The samples were examined using an FV10i fluorescence microscope (Olympus).

### MTS assay

Cell proliferation was evaluated by adding Celltiter 96^®^ Aqueous One Solution Reagent (Promega) to culture medium and incubated with cells at 37 °C and 5% CO_2_ for 1–4 h according to the manufacturer’s instructions.

### Real time PCR

Real time PCR was performed using 2 µl of cDNA, specific primers (BECN1, ATG7 and PIK3C3), SYBR Green qPCR (Roche, Basel, Switzerland) on a RochLightCycler^®^ 480II PCR (Roche Applied Science). cDNA was synthesized from 1 µg of total RNA using the iScript cDNA Synthesis kit (Bio-Rad, Veenendaal, The Netherlands) according to the manufacturer’s protocol. The expression levels of mRNA were normalized to β-actin expression.

### Statistical analysis

Analysis was performed using SPSS 17.0 statistical software. Measurement data are expressed as the mean ± standard deviation (*x* ± *s*). Comparisons between the two groups were tested by Student’s *t*-test. Univariate analysis of variance (ANOVA) was used as a homogeneity test of variance for comparisons among multiple groups, and the LSD *t*-test was used for pairwise comparisons among multiple sample means. Statistical significance was set at *p* < 0.05, unless otherwise indicated.

## Results

### *H. pylori* culture supernatant induces autophagy in human gastric cancer cells (SGC7901)

**Fig. 1:**
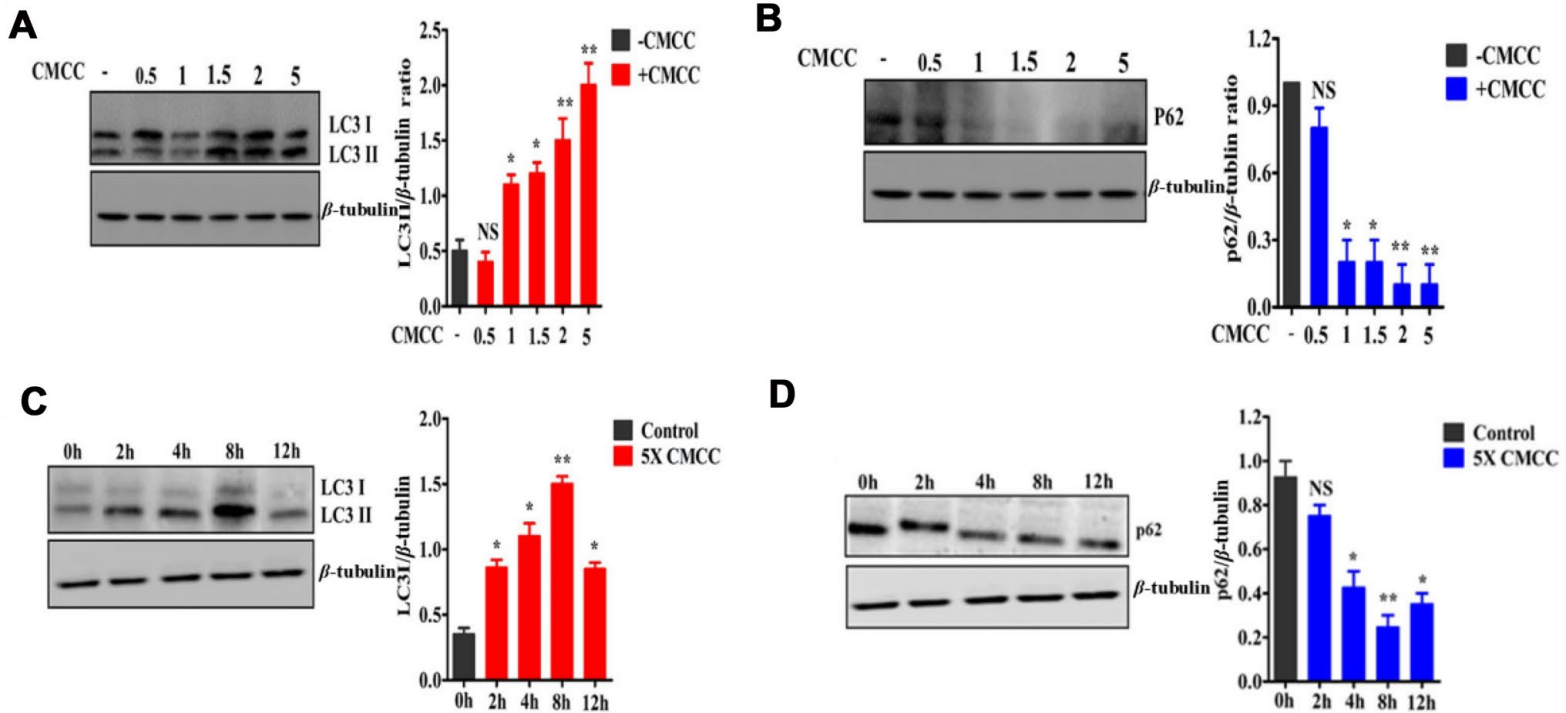
Expression of LC3II and p62 in SGC7901 cells treated with *H. pylori* culture supernatant at different concentrations and durations. **A** Western blotting was used to detect the expression of LC3. The results show that the transformation of LC3I into LC3II increased with increasing concentration and that the expression of LC3II/β-tubulin was enhanced. **B** Different concentrations of *H. pylori* supernatant were used to treat cells, and the expression of P62 gradually decreased. **C** Western blotting was used to detect the expression of LC3II. LC3II/*β*-tubulin levels increased with time, and autophagy peaked at 8 h. **D** Cells treated with concentrated *H. pylori* culture supernatant for 0, 2, 4, 8 and 12 h, and the expression of p62 gradually decreased. * means that a difference was statistically significant compared with the control group, *p* < 0.05; **p value < 0.01; NS, showed no significant difference compared with the control group, *p* > 0.05

We examined whether *H. pylori* culture supernatant could induce autophagy in human gastric cancer cells (SGC7901). After *H. pylori* culture supernatants were concentrated by ultrafiltration, we examined the effect of the supernatants on autophagy in SGC7901 cells. SGC7901 cells were treated with the supernatants diluted by 0.5-, 1-, 1.5-, 2-, and fivefold for 8 h. LC3I to LC3II conversion and expression of the ubiquitin-binding protein p62 were detected by Western blotting. We found that the *H. pylori* culture supernatants could induce autophagy in SGC7901 cells in a concentration-dependent manner (Fig. 1A, B).

Next, we tested the time course of the supernatants-induced autophagy in SGC7901 cells. After cells were treated with fivefold dilution of *H. pylori* culture supernatant for 0, 2, 4, 8 and 12 h, the protein levels of LC3II and p62 were detected by Western blotting. We found that the ratios of LC3II to β-tubulin were gradually increased and the protein levels of p62 were gradually decreased at 2 h, and peaked at 8 h (*p* < 0.05) (Fig. 1C, D). These results suggest that the *H. pylori* supernatant induce autophagy in SGC7901 cells.

### Human gastric cancer cell (SGC7901) death induced by VacA is dependent on autophagy

**Fig. 2:**
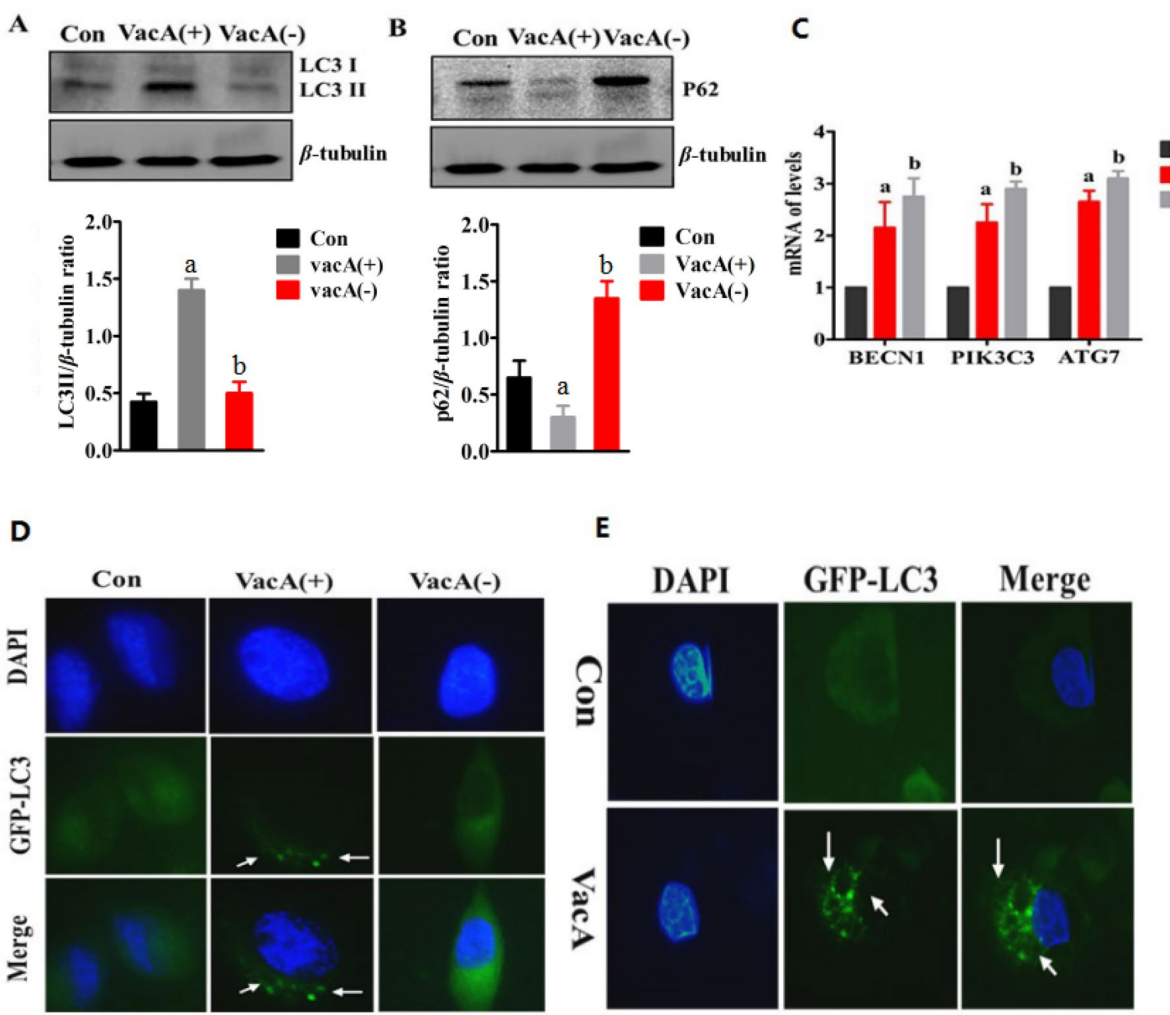
Autophagy after treatment with *H. pylori* culture supernatant or the VacA protein in SGC7901 cells. **A** After SGC901 cells were treated with culture supernatant, LC3II expression was detected by Western blotting. The results showed that the conversion of LC3I to LC3II was decreased and the LC3II/β-tubulin ratio was decreased, b *p* < 0.05. **B** Western blotting was used to detect the expression of P62. The results showed that the P62/β-tubulin ratio was increased, b *p* < 0.05. **C** After cells were treated with *H. pylori* culture supernatant or the VacA protein for 8 h, the expression levels of BECN1, PI3KC3 and ATG7 were enhanced, a *p* < 0.05 and b *p* < 0.05. **D** When concentrated *H. pylori* culture supernatant containing the VacA protein was used to treat stable GFP-LC3-expressing cells, a significantly increased number of GFP-LC3 particles were observed. **E** After purified VacA protein was used to treat GFP-LC3-expressing cells, the number of GFP-LC3 particles was increased, a *p* < 0.05 and b *p* < 0.05

To verify the role of the VacA protein in SGC7901 cells, we removed the VacA protein by immunoprecipitation using anti-VacA antibodies. After the VacA protein was removed from the supernatant, the LC3II/β-tubulin ratio was significantly reduced, whereas the p62 protein expression was simultaneously increased (Fig. 2A, B). These results indicate that the VacA protein in the *H. pylori* culture supernatant is responsible for induction of autophagy.

Next, the mRNA expression levels of autophagy-related genes (BECN1, PIK3C3, ATG7) were detected in SGC7901 cells treated with the *H. pylori* culture supernatant with or without the VacA protein. However, we found that supplementation with the *H. pylori* culture supernatant significantly up-regulated the mRNA expression of BECN1, PIK3C3 and ATG7 were in the presence or absence of VacA protein.

And we established a SGC7901 stable transfected cell line expressing GFP-LC3. We observed that the supernatant containing the VacA protein, but not the supernatant without the VacA protein, induced the formation of GFP-LC3 puncta (Fig. 2C).

To further prove the role of the VacA protein in autophagy, we purified the VacA protein by the ammonium sulfate precipitation. We found that supplementation with purified VacA markedly increased numbers of GFP-LC3 puncta. These results indicate that VacA plays a key role in autophagy in SGC7901 cells induced by *H. pylori*.

### Silencing Atg7 mRNA expression significantly reduced VacA protein-induced autophagy in SGC7901 cells

**Fig. 3:**
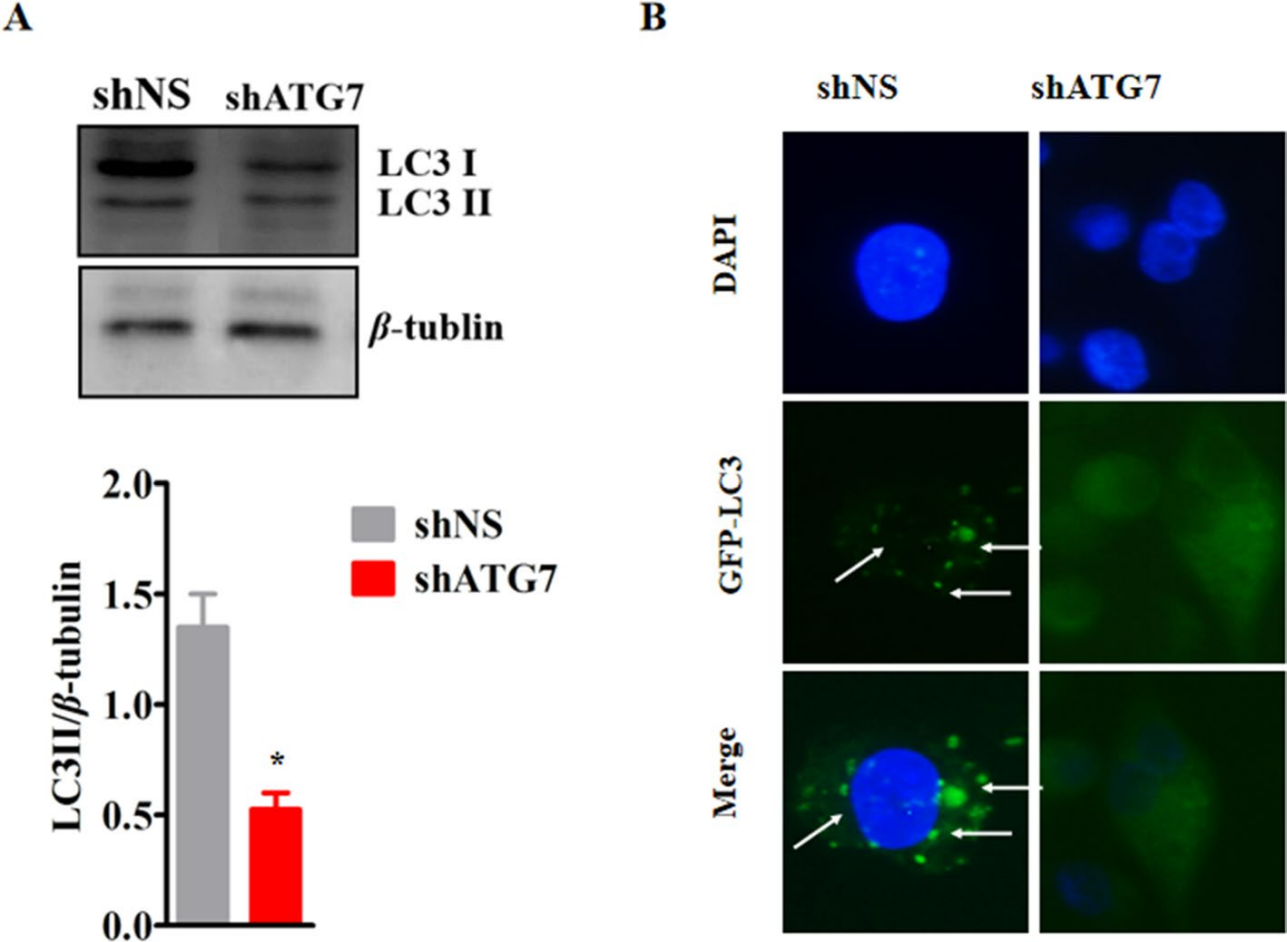
After Atg7 silencing, LC3 expression in SGC7901 cells was detected**.** **A** After Atg7 silencing and 8 h of treatment with VacA, the expression of LC3 in SGC7901 cells was detected by Western blotting. The conversion of LC3I to LC3II was decreased, and the LC3II/β-tubulin ratio was decreased, *p* < 0.05. **B** After Atg7 silencing and 8 h of treatment with VacA, GFP-LC3 particles in stable transfer cell lines were decreased under a fluorescence microscope

To verify that VacA protein-mediated autophagy occurred through the classical autophagy pathway, we silenced ATG7 mRNA expression in SGC7901 cells. We added the VacA protein for 8 h, detected LC3I/II protein expression and GPF-LC3 particles in the stable GFP-LC3-expressing cell line. After ATG7 silencing, the LC3II/bet-tubulin ratio was significantly reduced in SGC7901 cells, and the numbers of GFP-LC3 puncta were also significantly decreased in stable GPF-LC3-expressing cells (Fig. 3A, B). These results confirm that the autophagy induced by the VacA protein is carried out through the classical autophagy pathway.

### VacA increased the level of ROS and induced autophagy in SGC7901 cells

**Fig. 4:**
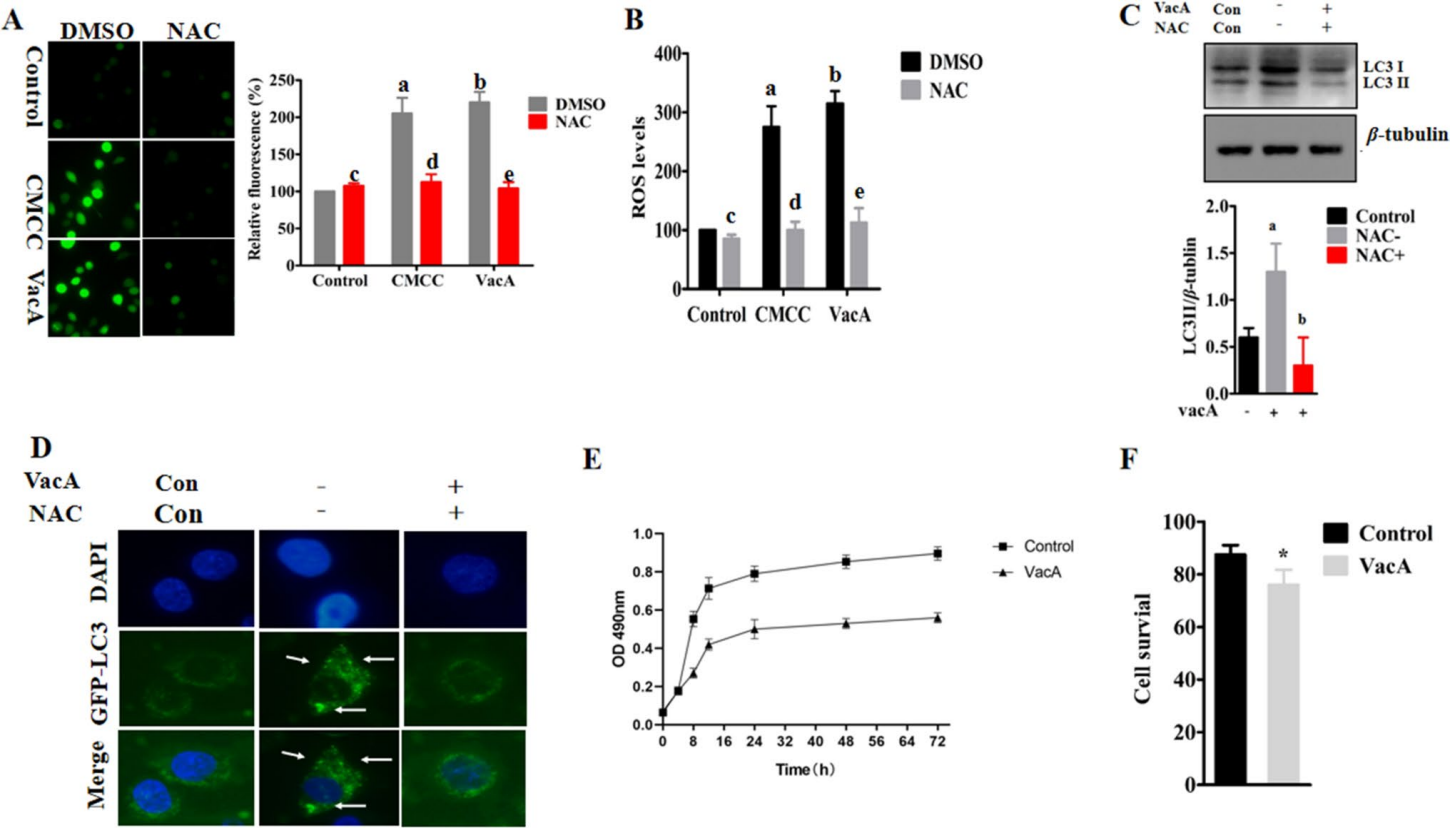
*H. pylori* culture supernatant or VacA protein treatment increased ROS levels and autophagy in SGC7901 cells. **A** ROS levels in SGC7901 cells were detected by H2DCF-DA staining. **B** After the treatment of cells with concentrated *H. pylori* culture supernatant or the VacA protein, ROS levels were quantitatively detected by H2DCF-DA staining with a microplate reader. Levels of significance are indicated as follows: a *p* < 0.05 vs the control group, b *p* < 0.05 vs the control group, c *p* < 0.05 vs the control group, d *p* < 0.05 vs the CMCC group, e *p* < 0.05 vs the VacA group. **C** After VacA and NAC were added to cells and incubated for 8 h, LC3II expression was detected by Western blotting. The results showed that the conversion of LC3I to LC3II was decreased after the addition of NAC and that the LC3II/β-tubulin ratio was significantly decreased, *p* < 0.05. **D** The VacA protein was used to treat a stable GFP-LC3-expressing cell line for 8 h, and an increased number of GFP-LC3 particles was observed under a fluorescence microscope, while a decreased number of GFP-LC3 particles was observed after the addition of NAC. **E** After the VacA protein was used to treat SGC7901 cells, an MTS kit was used to detect cell proliferation. The results showed that after treated with VacA for 8 h, cell proliferation was inhibited. **F** The survival rate of SGC7901 cells was significantly decreased after 12 h of treatment, as shown by trypan blue staining, *p* < 0.05

ROS levels were detected using 2,7-dichlorodihydrofluorescein diacetate (H2DCF-DA) in SGC7901 cells. Supplementation with the *H. pylori* culture supernatant or purified VacA protein significantly increased intracellular ROS levels (Fig. 4A). To further verify the experimental results, ROS levels were quantitatively detected with the dye H2DCF-DA, and the results were consistent with those of H2DCF-DA staining (Fig. 4B). After the addition of N-acetyl-l-cysteine (NAC, 5 mM), ROS levels were significantly decreased in the cells treated with the supernatant or the VacA protein (*p* < 0.05) (Fig. 4A, B). These results suggest that the VacA protein can increase ROS levels in gastric cancer cells.

To determine whether ROS are involved in autophagy induced by VacA, we inhibited ROS formation by pre-treatment of NAC. We found that the addition of NAC significantly decreased the LC3I/II ratio in SGC7901 cells treated by VacA (Fig. 4C). And NAC markedly reduced the numbers of GFP-LC3 puncta in stable GPF-LC3-expressing cells treated by VacA (Fig. 4D). These results suggest that the autophagy induced by VacA is mediated by ROS.

Then we determined the cell viability using MTS method. We found that supplementation with VacA inhibited cell proliferation (Fig. 4F). Next, we tested the survival rate of SGC7901 cells by trypan blue staining. Compared with that of the control group, the survival rate of SGC7901 cells in the VacA group was decreased (Fig. 4F).

## Discussion

Epidemiological studies have shown that gastric cancer is closely related to *H. pylori* infection (Diaconu et al. 2017; Plummer et al. 2015; Hartgrink et al. 2009). Animal experiments have also confirmed that *H. pylori* infection can cause gastric cancer (Lee et al. 2003). However, in this study, our findings reveal that the autophagy which *H. pylori* VacA mediates inhibits the proliferation of SGC7901 gastric cancer cells.

Autophagy, known as type II programmed death, can be considered a mechanism to promote cell survival, while excessive autophagy causes cell death (Levine and Yuan 2005). Terebiznik et al. (2009) have first reported that gastric epithelial cells infected with the HP60190 strain show VacA-mediated autophagy. Consistent with the results of many studies (Terebiznik et al. 2009; Tang et al. 2012; Butcher et al. 2017), we found that autophagic autophagy increased with increasing concentration of VacA. To verify that VacA is the key substance, we treated SGC7901 cells with or without VacA protein. Autophagy was significantly decreased after removal of VacA protein, which in turn verified that VacA is the major virulence factor of *H. pylori*-induced autophagy through Atg7.

ROS are highly reactive chemicals that can strongly oxidize molecules or ions, and can be produced in large quantities when the body is under stress, hunger, stimulated with drugs or under various diseases (Lim et al. 2001; Gong et al. 2012). Many studies have shown that the excessive accumulation of ROS can lead to cell lipid peroxidation, DNA damage, protein damage, and canceration (Yan et al. 2014; Possik et al. 2014; Bou et al. 2014). Wei et al. (2008) found that ROS can activate the inositol-dependent enzyme JNK1 and phosphorylate the BCL-2 protein, leading to dissociation of BCL-2 from the Beclin1 protein. Then Beclin1-VPS34-PI3KC3 complex is formed, thus activating autophagy. Zhang et al. (2009) found that H_2_O_2_ could activate the class III PI3K/Beclin1 signaling pathway, thus inducing autophagy by restraining the activity of Akt and mTOR (Smith et al. 2014; Circu and Aw 2010). VacA is known to cause mitochondrial damage. As mitochondrion is a main site of ROS generation, we speculate that VacA induces autophagy in SGC7901 cells through ROS. Indeed, the treatment of SGC7901 cells with VacA could significantly increase ROS expression. Furthermore, the addition of the antioxidant NAC reduced the autophagic activity induced by VacA. These results suggest that ROS play a decisive role in VacA-mediated autophagy.

ROS are closely related to tumor development. For instance, tumor cells are more sensitive to chemotherapy drugs with the increasing ROS level (Wu 2006). It has been reported that taxane and platinum drugs can produce ROS in large quantities during chemotherapy (Conklin 2004). In this study, the proliferation of SGC7901 cells is inhibited by addition of VacA protein, which is probably due to increased ROS formation. Whether pro-oxidant therapy can be used for the clinical prevention and treatment of tumors is worth further discussion.

According to our current study, the infection of gastric cancer cells with *H. pylori* vacuole toxin may have a protective effect on the human body, which is obviously different from the traditional view. But a recent prospective cohort study demonstrates that the total survival time of *H. pylori*-positive gastric cancer patients is longer than that of *H. pylori*-negative gastric cancer patients (Wang et al. 2013). In addition, Shimizu et al. (2017) reveal that *H. pylori* infection is related to tumor differentiation and lymph node metastasis. Gastric cancer patients infected with *H. pylori* had a better prognosis, but the specific mechanism was not clear. In our study, the proliferation of cancer cells was significantly inhibited by VacA treatment. We speculated that VacA-induced autophagy is probably responsible for gastric cancer cell death. This may be a reason that after formation, tumor differentiation and metastasis are increased compared to those in the *H. pylori*-negative group. However, the process of tumor growth is complicated and still needs to be further verified in vivo experiments.

In conclusion, the VacA protein inhibits SGC7901 cells proliferation through the ROS-mediated autophagy. This paper explains the role of VacA in the development of gastric cancer from a new perspective, and further study on autophagy is likely to provide new insight into the treatment of malignant tumors. The application of oxidants and autophagy activators may become a new strategy for the treatment of gastric cancer. However, how to accurately regulate ROS levels and autophagy to make them beneficial to the therapy is still a difficult problem at present.

## Data Availability

The datasets used and/or analyzed during the current study are available from the corresponding author on reasonable request.
